# Efficient Non-fullerene Organic Solar Cells Enabled by Sequential Fluorination of Small-Molecule Electron Acceptors

**DOI:** 10.3389/fchem.2018.00303

**Published:** 2018-07-26

**Authors:** Ruihao Xie, Lei Ying, Hailong Liao, Zhongxin Chen, Fei Huang, Yong Cao

**Affiliations:** State Key Laboratory of Luminescent Materials and Devices, Institute of Polymer Optoelectronic Materials and Devices, South China University of Technology, Guangzhou, China

**Keywords:** organic solar cells, non-fullerene, small molecule electron acceptors, fluorination, near-infrared absorption

## Abstract

Three small-molecule non-fullerene electron acceptors containing different numbers of fluorine atoms in their end groups were designed and synthesized. All three acceptors were found to exhibit relatively narrow band gaps with absorption profiles extending into the near-infrared region. The fluorinated analog exhibited enhanced light-harvesting capabilities, which led to improved short-circuit current densities. Moreover, fluorination improved the blend film morphology and led to desirable phase separation that facilitated exciton dissociation and charge transport. As a result of these advantages, organic solar cells based on the non-fullerene acceptors exhibited clearly improved short-circuit current densities and power conversion efficiencies compared with the device based on the non-fluorinated acceptor. These results suggest that fluorination can be an effective approach for the molecular design of non-fullerene acceptors with near-infrared absorption for organic solar cells.

## Introduction

Bulk heterojunction organic solar cells (OSCs) are a promising technology for solar energy collection and have attracted much interest owing to their unique advantages for the fabrication of lightweight and flexible devices (Li et al., 2016b, 2017a, 2018a,b; Zhao et al., 2016; Zhang et al., 2017b, 2018; Cheng et al., 2018; Hou et al., 2018; Zhang, 2018). Over the past several years, although fullerene derivatives have been extensively used as electron-acceptor materials for OSCs, their various intrinsic limitations, such as poor absorption in the visible-light region, a difficult-to-adjust molecular structure and morphological instability, have impeded the further development of OSCs (He and Li, 2011). To circumvent this constraint, considerable progress has been achieved recently due to the development of non-fullerene acceptors (NFAs) for high-performance non-fullerene OSCs, as NFAs have high absorption coefficients and suitable frontier molecular orbital energy levels that facilitate both the harvesting of solar photons and charge separation (Bin et al., 2016; Du et al., 2017; Fan et al., 2017; Kan et al., 2017b; Xu et al., 2017; Cui et al., 2018; Gao et al., 2018; Luo et al., 2018; Zhang et al., 2018; Zhu et al., 2018).

Typically, to achieve high photovoltaic performance of OSCs based on novel NFAs, much effort has been devoted to the use of advanced device structures and sophisticated film-processing techniques (Li et al., 2016a, 2017c; Meng et al., 2016; Bao et al., 2017; Kan et al., 2017a; Zhang et al., 2017a; Wu et al., 2018). It is well established that the light-harvesting capability of OSCs plays a critical role in their photovoltaic performance, as their power conversion efficiencies can be enhanced by expanding the absorption spectrum of the photoactive layer in the near-infrared (NIR) region. Therefore, NFAs with absorption spectra extending into the NIR region have been explored, especially with respect to their potential applications in semi-transparent organic photovoltaics and tandem OSCs (Li et al., 2017b; Yao et al., 2017, 2018). Furthermore, the fluorination of conjugated semiconductors has proved to be an effective synthetic strategy for developing efficient photoactive-layer materials for OSC applications. The introduction of fluorine atoms into small molecules or polymers can not only optimize their optical and electrical properties but also promote intermolecular interactions via the formation of non-covalent F···S and F···H bonds, resulting in enhanced charge mobility (Wang et al., 2013; Jo et al., 2015; Dai et al., 2017). More importantly, fluorination can be used to fine-tune the hydrophobicity and polarity of conjugated semiconductors, thus permitting control over the interfacial interactions in blend films (Pagliaro and Ciriminna, 2005). The combination of these advantages leads to improved film morphology with appropriate phase domains and larger interfacial areas, which facilitates exciton dissociation and charge transport and thus enhances the overall photovoltaic performance of OSCs.

In this work, we designed and synthesized a series of non-fullerene electron acceptors (BT-IC, BT-F, and BT-2F) with different numbers of fluorine atoms on their end groups. The strong intramolecular charge transfer between the electron-rich cores and electron-deficient end groups of these acceptors was found to result in intense absorption in the NIR region. The sequential fluorination of the end groups not only enhanced the light-harvesting capabilities of BT-F and BT-2F but also simultaneously increased their electron mobilities, leading to a higher short-circuit current density (*J*_SC_). More importantly, the fluorinated acceptors exhibited more favorable phase separation after blending with a medium-band-gap conjugated polymer. The combination of these phenomena led to improved short-circuit current density and thus enhanced the photovoltaic performance of the resulting devices.

## Experimental

### Instrumentation

^1^H and ^13^C NMR were characterized with Bruker-500 spectrometer in deuterated chloroform solution at 298 K. Chemical shifts were recorded as δ values (ppm) with the internal standard of tetramethylsilane (TMS). Mass spectra were collected on a MALDI Micro MX mass spectrometer, or an API QSTAR XL System. Number-average (*M*_n_) and polydispersity index (PDI) were determined on a Polymer Laboratories PL-GPC 220 using 1,2,4-trichlorobenzene as eluent at 150°C vs. polystyrene standards. Thermogravimetric analyses (TGA) were performed on a Netzsch TG 209 under nitrogen at a heating rate of 10°C min^−1^. Differential scanning calorimetry (DSC) was performed on a Netzsch DSC 204 under nitrogen flow at heating/cooling rates of 10/10°C min^−1^. The absorption coefficients of films are calculated by dividing the film thickness with the maximum absorption peak. The thin films with thickness of about 100 nm (measured by the profilometer) is spin-coated from chloroform solution on the top of quartz. Then the absorption spectra of these films were recorded by a HP 8453 spectrophotometer. Cyclic voltammetry (CV) was performed on a CHI600D electrochemical workstation with a glassy carbon working electrode and a Pt wire counter electrode at a scanning rate of 50 mV s^−1^ against an Ag/Ag^+^ reference electrode with a nitrogen saturated anhydrous solution of tetra-*n*-butylammonium hexafluorophosphate in acetonitrile (0.1 mol L^−1^). Atomic force microscopy (AFM) measurements were carried out using a Digital Instrumental DI Multimode Nanoscope III in a taping mode. TEM images were characterized with a JEM-2100F instrument.

### Photovoltaic device fabrication

The non-fullerene organic solar cells with a conventional device structure of ITO/PEDOT:PSS/active layer/PFN-Br/Ag were fabricated. Here PFN-Br represents poly[(9,9-bis(3′-((*N*,*N*-dimethyl)-*N*-ethylammonium)-propyl)-2,7-fluorene)-*alt*-2,7-(9,9-dioctylfluorene)] dibromide, which functioned as the cathode interlayer to facilitate electron extraction from the active layer. Before fabrication of the device, the indium tin oxide (ITO)-coated glass substrates were cleaned by ultrasonic treatment in deionized water, acetone, isopropyl alcohol, and dried in oven at 80°C for 12 h before used. After PEDOT:PSS (30 nm) layer was spin coated onto the substrate, and dried at 150°C for 15 min in air. Then, the ITO substrates were transferred into a nitrogen protected glovebox where the H_2_O concentration is ≤ 0.5 ppm and O_2_ concentration is ≤ 20 ppm. The thin film of active layer was spin-coated from a solution of PTZPF:non-fullerene acceptor blend in chlorobenzene. A thin PFN-Br layer (5 nm) was then spin coated onto the active layer as the cathode interface layer. The substrates were then transferred to a vacuum thermal evaporator, followed by deposition of the Ag cathode at a pressure of 2 × 10^−7^ Torr through a shadow mask. Before the *J-V* test, a physical mask with an aperture with precise area of 0.04 cm^2^ was used to define the device area. The *J-V* curves were measured on a computer-controlled Keithley 2,400 source meter under 1 sun, the AM 1.5 G spectra came from a class solar simulator (Enlitech, Taiwan), and the light intensity was 100 mWcm^−2^ as calibrated by a China General Certification Center-certified reference monocrystal silicon cell (Enlitech). The external quantum efficiency (EQE) spectra measurements were performed on a commercial QE measurement system (QE-R3011, Enlitech).

### Materials

**Figure 1:**
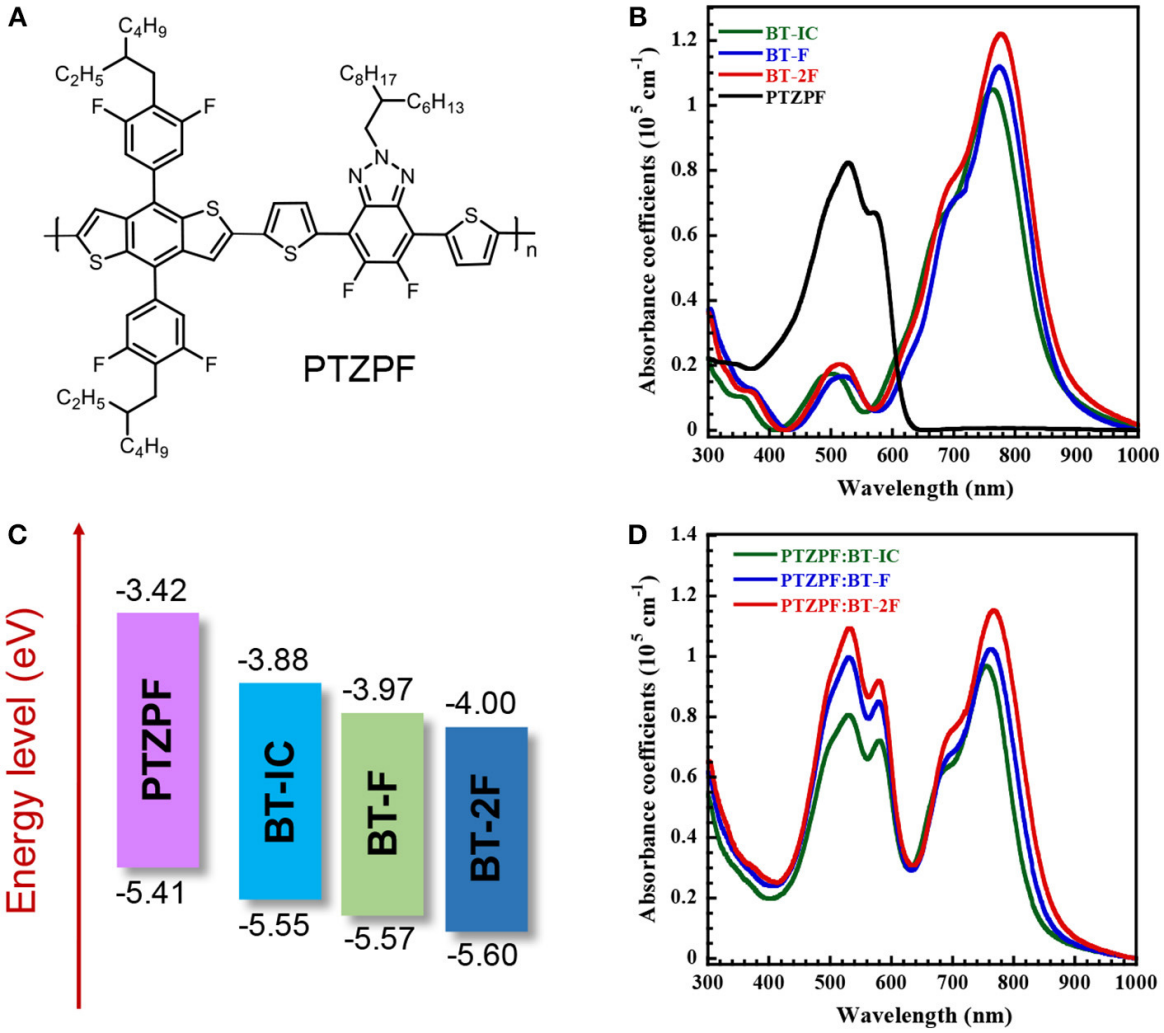
**(A)** Chemical structure of PTZPF; **(B)** UV–vis absorption spectra and **(C)** energy level diagrams of BT-IC, BT-F, BT-2F, and PTZPF; **(D)** UV–vis spectra of PTZPF:NFA blend films.

The monomers of thieno[3′,2′:4,5] cyclopenta[1,2-*b*] thieno[2″,3″:3′,4′] cyclopenta[1′,2′:4,5] thieno[2,3-*f*] [1] benzothiophene-2,8-dicarboxaldehyde, 5,11-bis[(2-ethylhexyl)oxy] -4,4,10,10-tetrakis(4-hexylphenyl)-4,10-dihydro **(1)** were synthesized according to the reported procedures (Li et al., 2017b). And the donor polymer PTZPF was synthesized via Stille polymerization (Scheme S1, supporting information, SI, with molecular structure shown in Figure 1A). 2-(3-Oxo-2,3-dihydro-1*H*-inden-1-ylidene)malononitrile (**2**), a mixture of 2-(5-fluoro-3-oxo-2,3-dihydro-1*H*-inden-1-ylidene)malononitrile and 2-(6-fluoro-3-oxo-2,3-dihydro-1*H*-inden-1-ylidene)malononitrile (**3**) and 2-(5,6-difluoro-3-oxo-2,3-dihydro-1*H*-inden-1-ylidene)malononitrile (**4**) were obtained from commercial sources and used without further purification. The small-molecule acceptors were prepared as the following procedures as below.

**Scheme 1:**
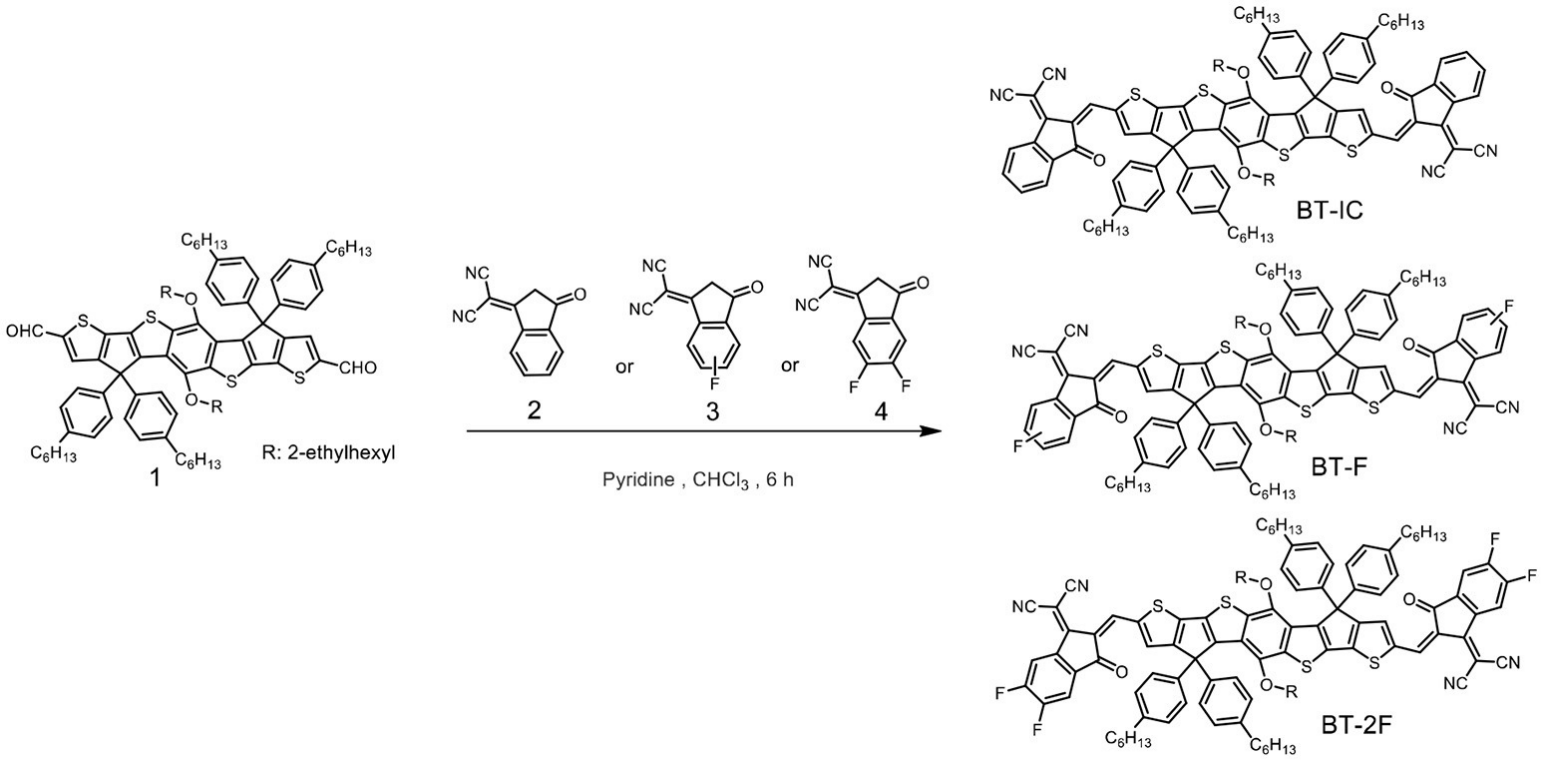
Synthetic route of the small-molecule non-fullerene acceptors.

#### Synthesis of BT-IC

2-(3-Oxo-2,3-dihydro-1*H*-inden-1-ylidene)malononitrile (**2**) (194.2 mg, 1.0 mmol) was added into the mixture of thieno[3′,2′:4,5] cyclopenta[1,2-*b*] thieno[2″,3″:3′,4′] cyclopenta[1′,2′:4,5] thieno[2,3-*f*] [1] benzothiophene-2,8-dicarboxaldehyde, 5,11-bis[(2-ethylhexyl)oxy] -4,4,10,10-tetrakis(4-hexylphenyl)-4,10-dihydro (**1**) (133.2 mg, 0.1 mmol) in chloroform (50 mL) with pyridine (1 mL). The reactant was refluxed for 6 h under nitrogen atmosphere. After cooling to room temperature, the reactant was poured into methanol and the precipitate was filtered off. The crude product was purified by silica gel using a mixture of hexane/dichloromethane as the eluent to give a blue black powder (121.2 mg, 73%). ^1^H NMR (400 MHz, CDCl_3_, δ): 8.79 (s, 2H), 8.64 (m, 2H), 7.87 (m, 2H), 7.70 (m, 4H), 7.48 (s, 2H), 7.31 (m, 8H), 7.08 (m, 8H), 3.48 (t, 4H), 2.57 (t, 8H), 1.60-1.53 (m, 2H), 1.35-1.28 (m, 48H), 0.96 (t, 6H), 0.87 (m, 18H). ^13^C NMR (100 MHz, CDCl_3_, δ): 188.56, 164.46, 160.31, 157.20, 153.89, 146.22, 142.16, 142.14, 140.67, 139.93, 138.69, 138.45, 138.23, 136.81, 135.89, 135.01, 134.30, 128.51, 128.50, 128.31, 125.25, 123.61, 121.32, 114.81, 68.40, 63.94, 39.38, 35.56, 31.71, 31.26, 31.24, 29.54, 29.21, 28.77, 23.34, 22.68, 22.59, 14.21, 14.10, 10.78. MS (MALDI-TOF) calcd for C_110_H_114_N_4_O_4_S_4_, 1684.386; found, 1683.622.

#### Synthesis of BT-F

A similar procedure was followed as that described for **BT-IC**, a mixture of 2-(5-fluoro-3-oxo-2,3-dihydro-1*H*-inden-1-ylidene)malononitrile and 2-(6-fluoro-3-oxo-2,3-dihydro-1*H*-inden-1-ylidene)malononitrile (**3**) (212.2 mg, 1.0 mmol) and thieno[3′,2′:4,5] cyclopenta[1,2-*b*] thieno[2″,3″:3′,4′] cyclopenta[1′,2′:4,5] thieno[2,3-*f*] [1]benzothiophene-2,8-dicarboxaldehyde, 5,11-bis[(2-ethylhexyl)oxy] -4,4,10,10-tetrakis(4-hexylphenyl)-4,10-dihydro (**1**) (133.2 mg, 0.1 mmol) were used. **BT-F** was obtained as a blue black solid (149.2 mg, 88.0 %). ^1^H NMR (400 MHz, CDCl_3_, δ): 8.79 (s, 2H), 8.68 (m, 0.5H), 8.36 (m, 1.5H),7.88 (m, 1.5H), 7.52 (m, 2.5H), 7.40 (m, 2H), 7.30 (m, 8H), 7.08 (m, 8H), 3.48 (m, 4H), 2.53 (m, 8H), 1.60-1.54 (m, 2H), 1.35-1.28 (m, 48H), 0.96 (t, 6H), 0.87 (m, 18H). ^13^C NMR (100 MHz, CDCl_3_, δ): 187.10, 167.69, 165.65, 165.19, 164.64, 164.61, 159.23, 158.89, 157.55, 157.50, 154.55, 154.39, 146.29, 142.28, 142.22, 142.19, 140.66, 140.62, 139.94, 139.88, 138.74, 138.69, 138.42, 138.29, 135.97, 135.79, 133.01, 128.50, 128.48, 128.39, 128.33, 127.76, 125.75, 125.67, 122.13, 121.67, 121.48, 121.15, 121.08, 114.81, 114.65, 114.50, 114.36, 112.89, 112.68, 110.75, 76.77, 69.09, 68.18, 63.95, 39.37, 35.55, 31.70, 31.25, 31.23, 29.56, 29.22, 28.78, 23.38, 22.71, 22.60, 14.22, 14.11, 10.80. MS (MALDI-TOF) calcd for C_110_H_112_F_2_N_4_O_4_S_4_, 1720.367; found, 1719.595.

#### Synthesis of BT-2F

A similar procedure was followed as that described for **BT-IC**, 2-(5,6-difluoro-3-oxo-2,3-dihydro-1*H*-inden-1-ylidene)malononitrile (**4**) (230.2 mg, 1.0 mmol) and thieno[3′,2′:4,5] cyclopenta[1,2-*b*] thieno[2″,3″:3′,4′] cyclopenta[1′,2′:4,5] thieno[2,3-*f*] [1] benzothiophene-2,8-dicarboxaldehyde, 5,11-bis[(2-ethylhexyl)oxy] -4,4,10,10-tetrakis(4-hexylphenyl)-4,10-dihydro (**1**) (133.2 mg, 0.1 mmol) were used. **BT-2F** was obtained as a black solid (138.6 mg, 80.0 %). ^1^H NMR (400 MHz, CDCl_3_, δ): 8.78 (s, 2H), 8.52 (m, 2H), 7.64 (t, 2H), 7.50 (s, 2H), 7.31 (dd, 8H), 7.08 (d, 8H), 3.49 (t, 4H), 2.57 (t, 8H), 1.60-1.54 (m, 2H), 1.36-1.28 (m, 48H), 0.96 (t, 6H), 0.86 (m, 18H). ^13^C NMR (100 MHz, CDCl_3_, δ): 186.22, 164.72, 160.58, 158.12, 155.49, 146.38, 142.47, 142.28, 140.62, 139.08, 138.95, 138.75, 138.35, 136.53, 136.03, 135.70, 134.62, 128.56, 128.48, 128.36, 120.53, 115.13, 114.39, 112.46, 68.67, 63.78, 39.40, 35.56, 31.72, 31.26, 31.24, 29.54, 29.21, 28.77, 23.34, 22.68, 22.59, 14.21, 14.10, 10.76. MS (MALDI-TOF) calcd for C_110_H_110_F_4_N_4_O_4_S_4_, 1756.348; found, 1755.581.

## Results and discussion

### Synthesis and characterization

The synthesis of the target compounds BT-IC, BT-F, and BT-2F is outlined in Scheme 1. These small-molecule acceptors were prepared via Knoevenagel condensation between thieno[3′,2′:4,5] cyclopenta[1,2-*b*] thieno[2″,3″:3′,4′] cyclopenta[1′,2′:4,5]thieno[2,3-*f*] [1] benzothiophene-2,8-dicarboxaldehyde, 5,11-bis[(2-ethylhexyl)oxy] -4,4,10,10-tetrakis(4-hexylphenyl)-4,10-dihydro (**1**) and 2-(3-oxo-2,3-dihydro-1*H*-inden-1-ylidene)malononitrile (**2**) or its fluorinated derivatives at 60°C in the presence of a catalytic amount of pyridine. It is worth noting that the monofluorinated compound **3** consisted of two regioisomers, namely, 2-(5-fluoro-3-oxo-2,3-dihydro-1*H*-inden-1-ylidene)malononitrile and 2-(6-fluoro-3-oxo-2,3-dihydro-1*H*-inden-1-ylidene)malononitrile. As these two isomers have very similar molecular structures and polarities, they could not be separated, and thus the resulting BT-F was obtained as a mixture of isomers. All three acceptors exhibited good solubility in typical organic solvents, such as chloroform, chlorobenzene and *ortho*-dichlorobenzene, at room temperature. The chemical structures of the three acceptors were confirmed by nuclear magnetic resonance spectroscopy and mass spectrometry (Figures S7–S12).

The thermal properties of these resulting NFAs were evaluated by thermogravimetric analysis and differential scanning calorimetry (DSC) under a nitrogen atmosphere (Figures S1, S2,). All of these NFAs exhibited excellent thermal stabilities with onset decomposition temperatures (*T*_d_) higher than 310°C. The DSC curves were obtained by heating from 30 to 250°C in the second heating/cooling cycle. It was found that BT-IC exhibited a melting peak at 127°C, whereas no phase-transition signals were observed during the DSC measurements of the other two materials.

### Optical, electrochemical, and electron-transport properties

Figure 1B shows the UV–vis absorption spectra of thin films of the donor polymer and acceptor molecules. All three NFAs showed similar absorption profile cut-offs in the NIR region (up to 866 nm) in the solid state. Such absorption profiles are complementary with a medium band gap conjugated polymer, namely PTZPF, which has the absorption onset of 620 nm (Figure 1D). Note that the complementary absorption is beneficial for the harvesting of solar photons to achieve a high short-circuit current density. The fluorinated small molecules BT-F and BT-2F exhibited slightly red-shifted absorption edges compared with BT-IC. An optical band gap of 1.43 eV was obtained for BT-IC, which slightly decreased to 1.42 and 1.41 eV for BT-F and BT-2F, respectively. Importantly, the absorption coefficients were slightly enhanced from 1.05 × 10^5^ cm^−1^ for BT-IC to 1.12 × 10^5^ cm^−1^ and 1.22 × 10^5^ cm^−1^ for BT-F and BT-2F, respectively (Figure 1B), indicating that the introduction of fluorine atoms into these acceptors enhanced their light-harvesting ability through improved intermolecular interactions (Yang et al., 2013; Wolf et al., 2015).

The electrochemical properties and energy levels of the polymer acceptors were investigated by cyclic voltammetry. Here we used the potential ferrocene/ferrocenium (Fc/Fc^+^) redox couple as the standard. Under the current measurement conditions, the potential of Fc/Fc^+^ couple was measured as 0.30 V regarding to the reference electrode. Assuming that the Fc/Fc^+^ redox couple has an absolute potential of −4.80 V to vacuum, the highest occupied molecular orbital energy levels (*E*_HOMO_) is calculated as *E*_*HOMO*_ = –e (*E*_ox_ + 4.80 – 0.30) (eV), and the lowest unoccupied molecular orbital energy levels (*E*_LUMO_) is calculated as *E*_LUMO_ = –e (*E*_red_ + 4.80 – 0.30) (eV). The energy level diagrams of all of the materials are depicted in Figure 1C and the corresponding electrochemical data are summarized in Table 1. The calculated *E*_LUMO_/*E*_HOMO_ values of BT-IC, BT-F and BT-2F were −3.88/−5.55 eV, −3.97/−5.57 eV and −4.00/−5.60 eV, respectively. Both the *E*_LUMO_ and *E*_HOMO_ levels of these acceptor molecules gradually decreased with the increasing number of fluorine atoms, indicating that fluorination of the end groups of small-molecule NFAs can effectively decrease the *E*_LUMO_ and *E*_HOMO_ levels owing to the strongly electron-withdrawing characteristics of fluorine atoms (Dutta et al., 2014). The *E*_LUMO_ and *E*_HOMO_ levels of the polymer donor PTZPF were −3.42 and −5.41 eV, respectively, which ensures an adequate driving force for efficient exciton dissociation (Scharber et al., 2010). The charge carrier mobilities of the pure films of acceptors were measured by the space-charge-limited current (SCLC) method using the Mott–Gurney equation (Figure S5). The measurements are carried out by fabricating electron-only devices with architecture of ITO/Ag/active layer/Ag structure. The pure film based on BT-2F exhibited the electron mobility (μ_e_) of 9.64 × 10^−5^ cm^2^ V^−1^ s^−1^, which is higher than those of BT-F (μ_e_ = 5.53 × 10^−5^ cm^2^ V^−1^ s^−1^) and BT-IC (μ_e_ = 2.28 × 10^−5^ cm^2^ V^−1^ s^−1^).

**Table 1:** Optophysical and electrochemical properties of active layer materials.

**Materials**	**λ_abs_ (nm) film**	**λ_abs_ (nm) onset**	**^a^*E*_g_°^pt^ (eV)**	***E*_ox_ (V)**	***E*_red_ (V)**	**^b^*E*_HOMO_ (eV)**	***^c^E*_LUMO_ (eV)**
BT-IC	760	866	1.43	1.05	−0.62	−5.55	−3.88
BT-F	774	872	1.42	1.07	−0.53	−5.57	−3.97
BT-2F	778	878	1.41	1.10	−0.50	−5.60	−4.00
PTZPF	528	620	2.00	0.91	−1.08	−5.41	−3.42

a *Calculated from the onset of UV-vis absorption as pristine thin films*;

b *E_HOMO_ = –e (E_ox_ + 4.50) (eV)*;

c *E_LUMO_ = –e (E_red_ + 4.50)*.

### Photovoltaic performances

**Figure 2:**
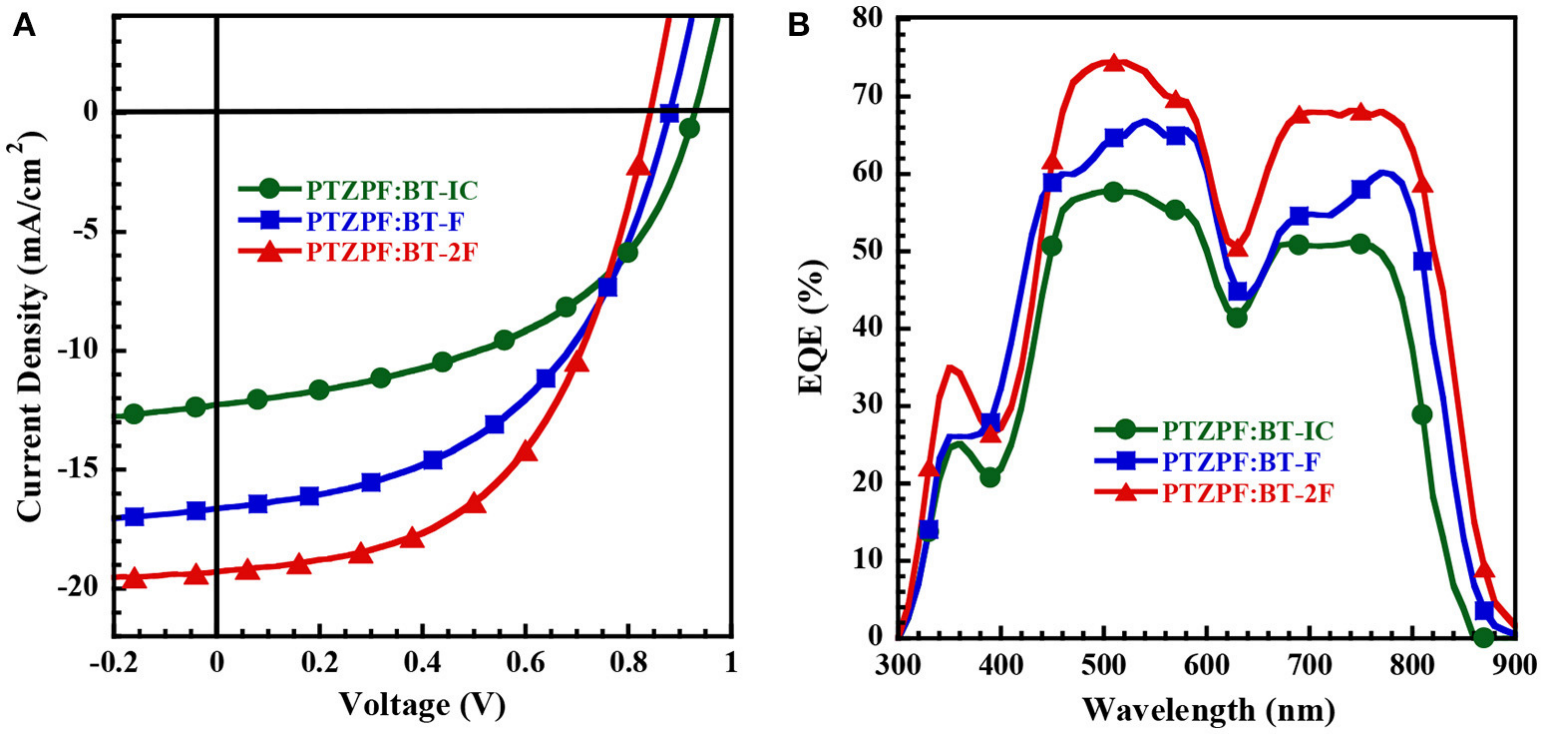
**(A)** *J*–*V* curves and **(B)** EQE spectra of OSC devices measured under AM 1.5 G illumination at 100 mW cm^−2^.

To elucidate the effects of fluorination on the photovoltaic properties, OSC devices were fabricated using PTZPF as the electron-donor material and BT-IC, BT-F, or BT-2F as the electron-acceptor material. The devices were fabricated with the conventional configuration of ITO/PEDOT:PSS/active layer/PFN-Br/Ag, and the device performances were measured under simulated AM 1.5 G illumination at 100 mW cm^−2^. Poly[(9,9-bis(3'-((*N*,*N*-dimethyl)-*N*-ethylammonium)propyl)-2,7-fluorene)-*alt*-2,7-(9,9-dioctylfluorene)] dibromide (PFN-Br) was used as the cathode interfacial layer to facilitate charge carrier collection (Zhang et al., 2017c). The initial optimisation of device performance was carried out by screening the weight ratios of the donor:acceptor (D:A) blend, film thickness of the photoactive layers and the effects of additives to the processing solvents (Figure S6 and Table S1). All of the photoactive layers of the devices were processed under the optimized conditions, which consisted of a D:A weight ratio of 1:1, spin casting of the films from chlorobenzene containing 0.5 v/v% of 1-chloronaphthalene (CN) as additive, and annealing of the fabricated films at 120°C for 10 min. The current density–voltage (*J–V*) curves are presented in Figure 2A and the corresponding data are summarized in Table 2.

**Table 2:** Photovoltaic parameters of OSCs measured under AM1.5 Illumination at 100 mW cm^−2^.

**Active layer*^a^***	***V*_OC_ (V)**	***J*_SC_^b^(mA cm^−2^)**	***J*_SC, EQE_^c^(mA cm^–2^)**	**FF (%)**	**PCE (%)**
PTZPF:BT-IC	0.93	12.27	12.23	49.0	5.63 (5.45)^d^
PTZPF:BT-F	0.88	16.64	16.36	49.0	7.27 (7.00)
PTZPF:BT-2F	0.84	19.29	18.88	53.0	8.54 (8.50)

a *All of the blend films are processed by CB with 0.5 vol % CN and treated with 120°C for 10 min*;

b *Obtained from J–V measurements*;

c *Obtained from the integration of EQE spectra*;

d *Average values across more than 6 devices. Device structure: ITO/PEDOT:PSS/active layer/PFN-Br/Ag*.

Interestingly, the photovoltaic parameters of the resulting devices were strongly dependent on the number of fluorine substituents. The device based on BT-IC as the acceptor exhibited a moderate power conversion efficiency (PCE) of 5.63%, with an open-circuit voltage (*V*_OC_) of 0.93 V, a *J*_SC_ of 12.27 mA cm^−2^ and a fill factor (FF) of 49.0%. In contrast, the devices based on the fluorinated acceptors BT-F and BT-2F exhibited clearly enhanced PCE values of 7.27% (*V*_OC_ = 0.88 V, *J*_SC_ = 16.64 mA cm^−2^ and FF = 49.0%) and 8.54% (*V*_OC_ = 0.84 V, *J*_SC_ = 19.29 mA cm^−2^ and FF = 53.0%), respectively. It should be noted that despite the decrease in the *V*_OC_ of the resulting devices upon the incorporation of fluorine substituents into the acceptors, which is consistent with the down-shifted *E*_LUMO_ levels observed for BT-F and BT-2F (Brabec et al., 2010; Kang et al., 2012), the *J*_SC_ values were dramatically enhanced. The combination of these effects led to the clearly enhanced PCE values of the devices based on the fluorinated acceptors.

To investigate the obvious enhancement of *J*_SC_, we analyzed the absorption of PTZPF:BT-IC, PTZPF:BT-F and PTZPF:BT-2F blend films (Figure 1D). Similar to the absorption of neat films of BT-IC, BT-F, or BT-2F, the absorption coefficients of the PTZPF:BT-F and PTZPF:BT-2F blend films were both slightly higher than that of the PTZPF:BT-IC blend film, which can be correlated to the improved *J*_SC_ of the devices based on fluorinated acceptors. Furthermore, to confirm the accuracy of the observed *J*_SC_, we measured the external quantum efficiencies (EQEs) of the devices. It should be noted that the calculated *J*_SC_ values from the EQE spectra (Figure 2B) matched well with the *J*_SC_ values obtained from the *J*–*V* curves. The device based on BT-2F exhibited a stronger photocurrent response from 400 to 870 nm, with a maximum EQE of 75%, which was higher than those observed for the devices based on BT-F and BT-IC (Figure 2B).

### Charge generation, transport, and recombination

**Figure 3:**
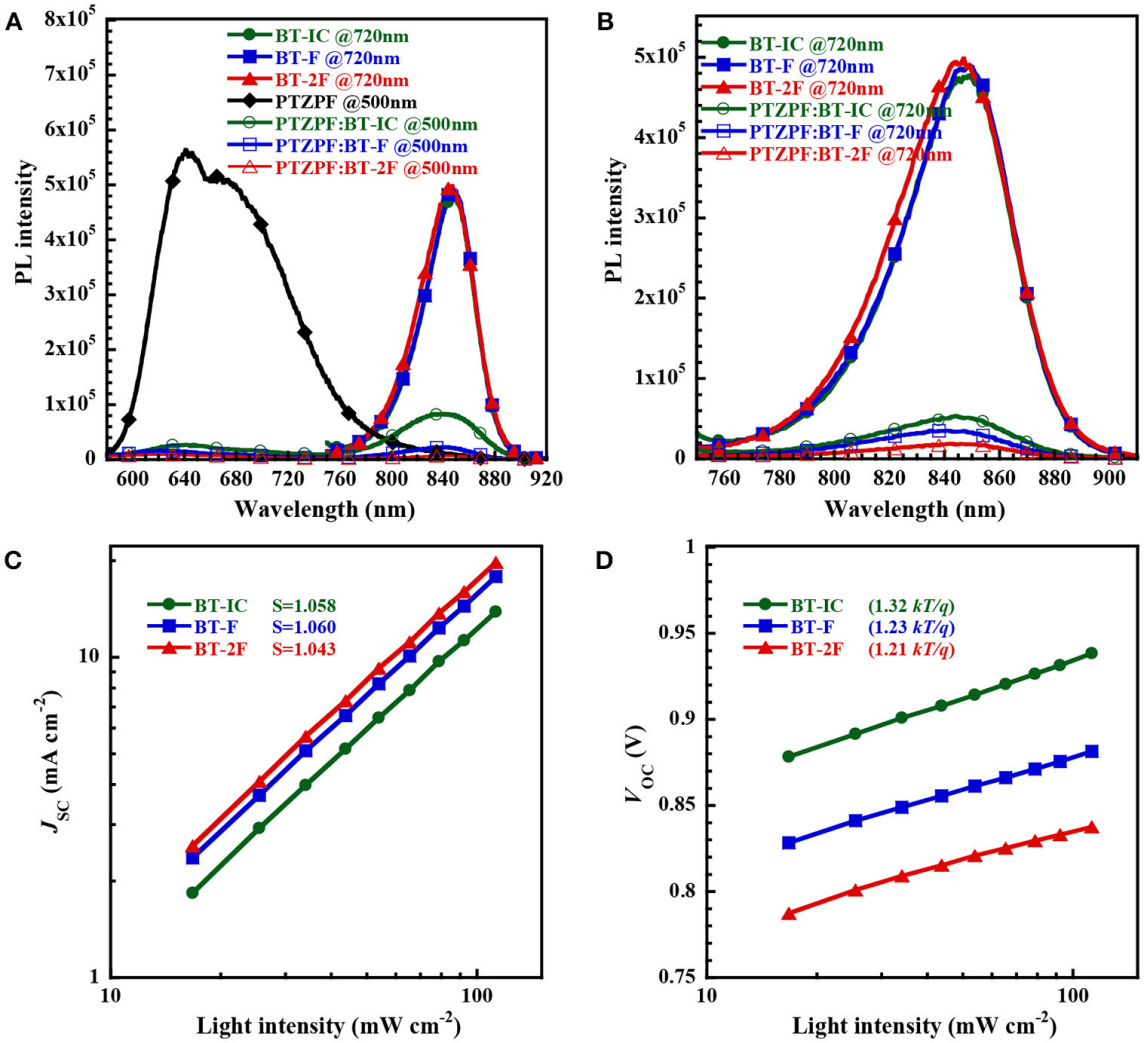
**(A,B)** Photoluminescence spectra of pristine donor films (excited at 500 nm), pristine acceptor films (excited at 720 nm) and corresponding blend films (excited at both 500 and 720 nm) with a film thickness of about 100 nm; **(C,D)** plots of (c) *J*_SC_ and (d) *V*_OC_ vs. light intensity for devices based on PTZPF:NFA blend films.

To study the charge generation process in the resulting bulk-heterojunction films, we measured the photoluminescence (PL) spectra of the neat and D:A blend films. The peak emission of the pure PTZPF film was located at 640 nm upon excitation at 500 nm, whereas the acceptor molecules BT-IC, BT-F, and BT-2F exhibited similar emission peaks at ~845 nm upon excitation at 720 nm. As shown in Figure 3A, the strong emission peak of BT-IC was clearly observed in the PTZPF:BT-IC blend film, indicating the low charge separation efficiency of the device based on BT-IC. In contrast, the PL emission of the neat films was effectively quenched in the PTZPF:BT-F and PTZPF:BT-2F blend films, indicating that exciton dissociation and charge transfer were remarkably enhanced by the introduction of fluorine atoms into the acceptor moiety. A similar phenomenon can be observed in Figure 3B, where the PL of BT-2F was quenched by 92.7% in the blend film, which was more pronounced than the quenching observed for the blend films based on BT-F (90.9%) or BT-IT (86.4%).

The *J*_SC_ and *V*_OC_ values of the devices were measured as a function of the light intensity (*P*_light_) to elucidate the charge recombination dynamics in the photoactive layer, as shown in Figures 3C,D, respectively. For organic solar cells, the power-law dependence of *J*_SC_ on the illumination intensity can generally be expressed as *J*_SC_^∝^(*P*_light_)^*S*^, where *S* is the exponential factor, which is close to unity when the bimolecular recombination in the device is weak (Kyaw et al., 2013; Lu et al., 2015). The extracted values of *S* were 1.058, 1.060 and 1.043 for the devices based on BT-IC, BT-F, and BT-2F, respectively, all of which were close to unity, indicating the weak bimolecular recombination in these devices (Yang et al., 2016). Based on the *V*_OC_-*P*_light_ plot, *V*_OC_ was plotted against the natural logarithm of *P*_light_ and the slope of n*kT*/*q* was calculated, where an n value of unity implies predominantly bimolecular recombination and an enhanced dependence of *V*_OC_ on *P*_light_ (2*kT*/*q*) indicates trap-assisted monomolecular recombination (Gasparini et al., 2016). The calculated slopes were 1.32, 1.23, and 1.21 *kT*/*q* for the devices based on BT-IC, BT-F and BT-2F, respectively. The smaller slope value for BT-2F than the others suggests less trap-assisted recombination, thus resulting in a higher FF value.

### Film morphology

**Figure 4:**
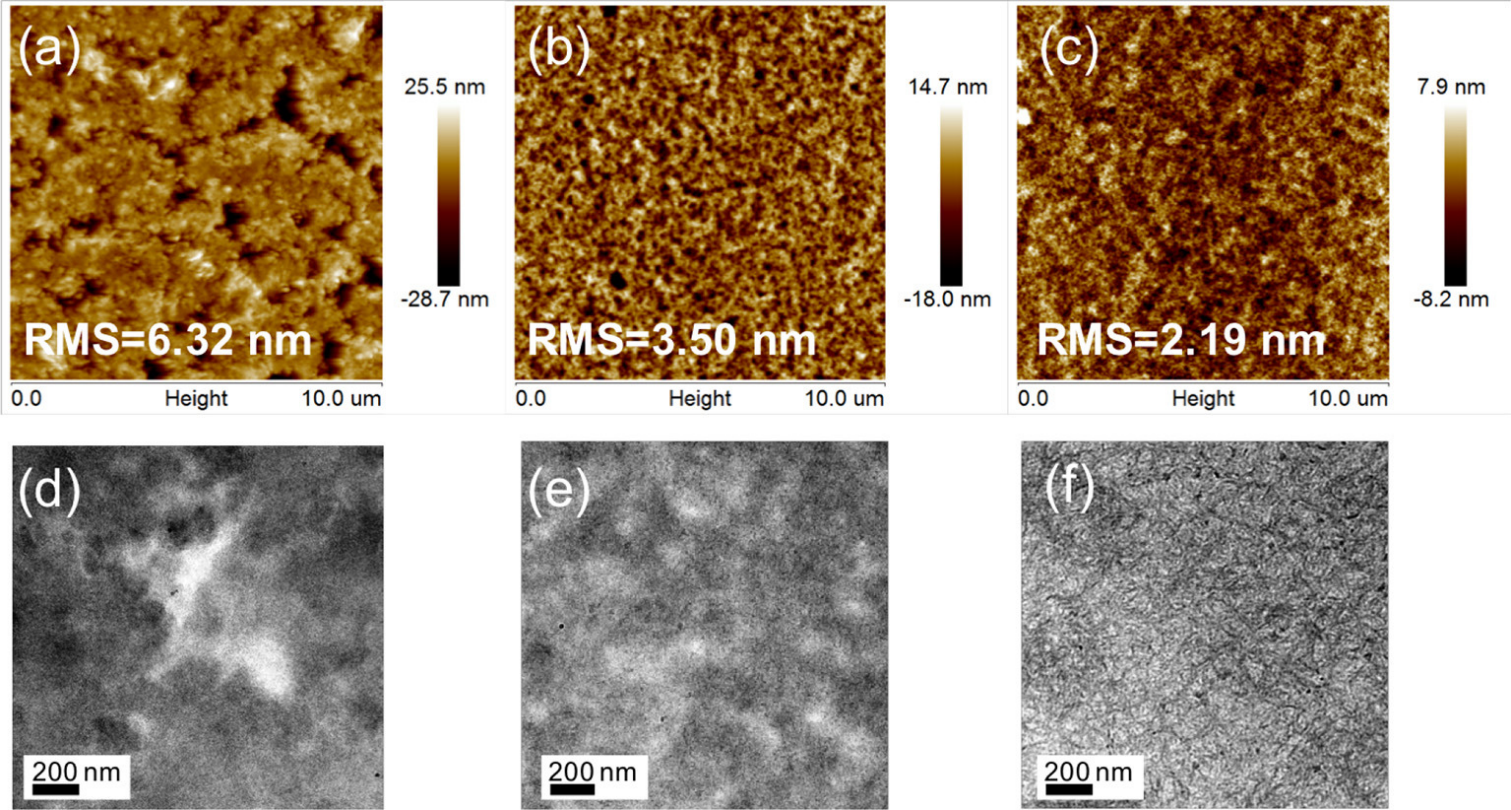
AFM height images (10 μm × 10 μm) of **(a)** PTZPF:BT-IC, **(b)** PTZPF:BT-F, and **(c)** PTZPF:BT-2F; TEM images of **(d)** PTZPF:BT-IC, **(e)** PTZPF:BT-F, and **(f)** PTZPF:BT-2F.

Tapping-mode atomic force microscopy (AFM) and transmission electron microscopy (TEM) measurements were performed to determine the influence of fluorination on the film morphology. Figures 4a–c shows topographical AFM images of active layers of the different NFAs. The PTZPF:BT-IC blend film contained large granular aggregates across the entire film with a root-mean-square (RMS) roughness of 6.32 nm (Figure 4a), whereas the blend films gradually became smoother as the number of fluorine atoms increased, with RMS roughness values of 3.50 nm and 2.19 nm for BT-F and BT-2F, respectively, suggesting that the incorporation of fluorine atoms led to a smoother film morphology. The phase separation of the blend films with different electron acceptors was also readily apparent from the TEM images. Figure 4d shows that the PTZPF:BT-IC blend film exhibited large-scale phase-separation features across the entire film, which is unfavorable for charge transfer at the donor–acceptor interface. Interestingly, the degree of phase separation of the blend films gradually decreased as the number of fluorine atoms increased. Consequently, the PTZPF:BT-2F blend film exhibited a smoother surface morphology with favorable phase separation (Figure 4f), which induced desirable exciton dissociation and thus simultaneously enhanced the *J*_SC_ and FF values.

## Conclusions

In summary, three NIR-absorbing electron acceptors containing different numbers of fluorine atoms were designed and synthesized. The results revealed that the fluorinated acceptors outperformed their non-fluorinated counterpart BT-IC. Sequentially increasing the number of fluorine atoms on the end groups of the acceptor molecules led to a dramatic improvement in the *J*_SC_ of the resulting photovoltaic devices. Non-fullerene OSCs based on the fluorinated acceptor BT-2F exhibited an improved PCE of 8.54% with a high *J*_SC_ of 19.29 mA cm^−2^, regarding to the device based on BT-IC (PCE = 5.63%, *J*_SC_ = 12.27 mA cm^−2^) that does not contain fluorine atom. The improved photovoltaic performances of devices based on fluorinated acceptors can be correlated to the broad absorption profile extending into the NIR region, favorable film morphology and efficient charge transfer. These results demonstrate that fluorination can be an effective technique in the design of efficient electron-acceptor materials.

## Author contributions

RX, LY and FH conceived the ideas and coordinated the work. RX and LY designed the donor polymer and the acceptor molecules. RX synthesized the polymer PTZPF and conducted the DSC, TGA, UV-vis, PL and cyclic voltammetric measurements. HL performed the device fabrication, the light intensity-dependent *J-V* characterization, and analyzed the data. ZC synthesized the acceptor molecules of BT-IC, BT-F, and BT-2F. RX and HL conducted the AFM and TEM measurements. RX, LY, FH, and YC contributed to manuscript preparation. All authors commented on the manuscript and approved for submission.

### Conflict of interest statement

The authors declare that the research was conducted in the absence of any commercial or financial relationships that could be construed as a potential conflict of interest.
