# Supplementary material for: Efficient Non-fullerene Organic Solar Cells Enabled by Sequential Fluorination of Small-Molecule Electron Acceptors
Source: Front Chem. 2018 Jul 26;6:303. doi: 10.3389/fchem.2018.00303 (PMC6071513; doi:10.3389/fchem.2018.00303)
Supplement: Supplementary file 1 [file Data_Sheet_1.DOCX]

Supplementary Material

Efficient Non-Fullerene Organic Solar Cells Enabled by Sequential Fluorination of Small Molecule Electron Acceptors

Ruihao Xie, Lei Ying^*^, Hailong Liao, Zhongxin Chen, Fei Huang* and Yong Cao

*** Correspondence:** Lei Ying: msleiying@scut.edu.cn

Fei Huang: msfhuang@scut.edu.cn

**Figure S1**. TGA curves of BT-IC, BT-F, BT-2F and PTZPF.

**Figure S2**. DSC curves of BT-IC, BT-F, BT-2F and PTZPF.

**Figure S3**. UV-vis absorption spectra of BT-IC, BT-F, BT-2F in chloroform solution.

**Figure S4**. Cyclic voltammograms of BT-IC, BT-F, BT-2F and PTZPF.

**Figure S5**. *J*^1/2-^*V* characteristics of pure films from SCLC (electron).

**Figure S6.**  *J*–*V* curves of OSC devices based on PTZPF:BT-IC (a), PTZPF:BT-F (b), and PTZPF:BT-2F (c) with various solvent additives.

**Table S1** Photovoltaic parameters of CB processed OSCs with the active layers processed by various solvent additives under AM1.5 Illumination at 100 mW/cm^2^

| Donor : Acceptor | Solvent  additive | *V*_OC_  (V) | *J*_SC_  (mA cm^-2^) | FF  (%) | PCE  (%) |
| --- | --- | --- | --- | --- | --- |
| PTZPF:BT-IC *^a^* | — | 0.95 | 12.13 | 42.0 | 4.84 |
|  | 0.5% DIO | 0.92 | 6.90 | 46.2 | 2.94 |
|  | 0.5% DBE | 0.96 | 6.86 | 47.9 | 3.15 |
|  | 0.5% CN | 0.93 | 12.27 | 49.0 | 5.63 |
| PTZPF:BT-F *^a^* | — | 0.88 | 15.33 | 47.8 | 6.61 |
|  | 0.5% DIO | 0.91 | 7.71 | 45.1 | 3.23 |
|  | 0.5% DBE | 0.88 | 12.35 | 54.0 | 5.99 |
|  | 0.5% CN | 0.88 | 16.64 | 49.0 | 7.27 |
| PTZPF:BT-2F *^a^* | — | 0.85 | 12.23 | 36.0 | 3.70 |
|  | 0.5% DIO | 0.84 | 17.34 | 38.2 | 5.60 |
|  | 0.5% DBE | 0.84 | 17.99 | 45.1 | 6.87 |
|  | 0.5% CN | 0.84 | 19.29 | 53.0 | 8.54 |

^a^ The Donor:Acceptor ratio is 1:1 and all of the blend films are treated with 120 ^o^C for 10 min.

**Scheme S1** Synthetic route of polymer PTZPF

**Synthesis of PTZPF.** 4,7-Bis(5-bromothiophen-2-yl)-5,6-difluoro-2-(2-hexyldecyl)-2H-benzo[*d*][1,2,3]triazole (**A**) (170.2 mg, 0.1mmol), (4,8-bis(4-(2-ethylhexyl)-3,5-difluorophenyl)benzo[1,2-*b*:4,5-*b*']dithiophene-2,6-diyl)bis(trimethylstannane) (**B**) (96.5 mg, 0.1 mmol) and Pd(PPh_3_)_4_ (7.0 mg) were dissolved in anhydrous chlorobenzene (1.5 mL) under a nitrogen atmosphere. The reaction mixture was heated at 140 °C with vigorous stirring for 48 h. Then, the bromo-terminal groups were capped with 2-(tributylstannyl)thiophene (0.1 mL), refluxing for 2 h. After that, the trimethylstannyl-terminal groups were capped with 2-bromothiophene (0.2 mL), refluxing for another 2 h. After cooling to room temperature, the polymer was precipitated from the solution into methanol and was collected by filtration and then successively purified by Soxhlet extraction with methanol, hexane and chloroform. The chloroform fraction was purified by passing it through a short silica gel column and the final product was obtained by precipitated into methanol again. The resulting polymer was then dried under vacuum for 1 day to get the polymer **PTZPF** (105 mg, 88.7 %). (*M*_n_ = 13.2 kg mol^-1^, *PDI* = 2.2).


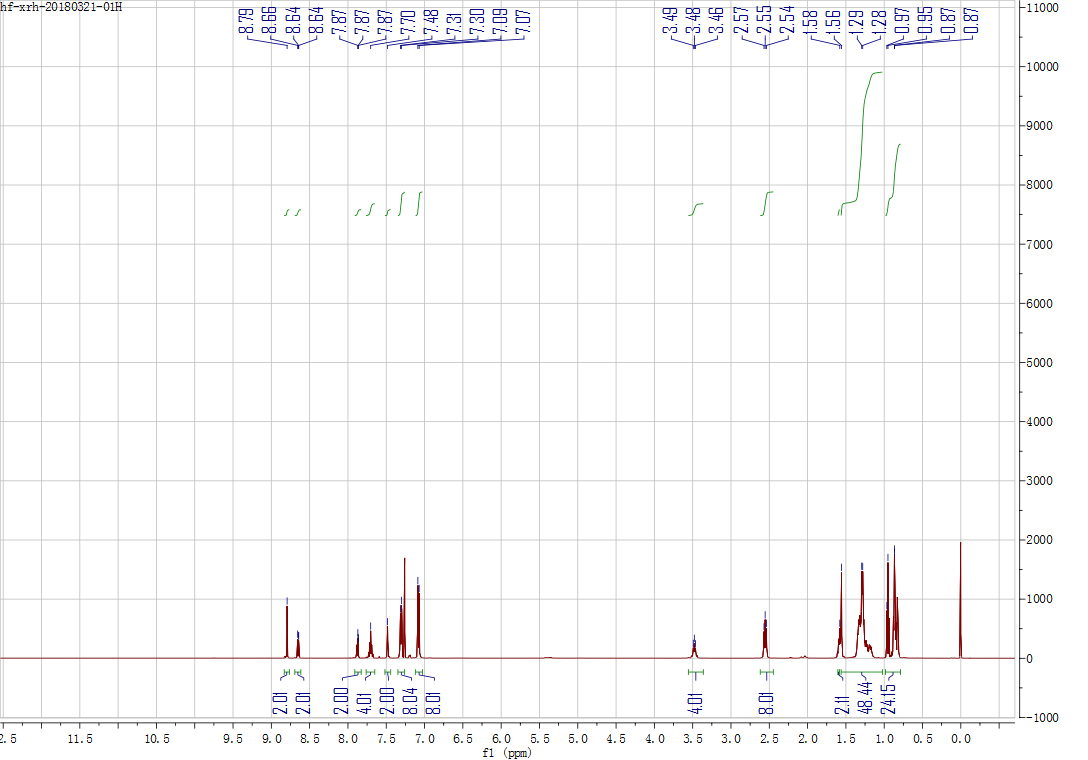


**Figure S7**. ^1^H NMR spectrum of BT-IC.


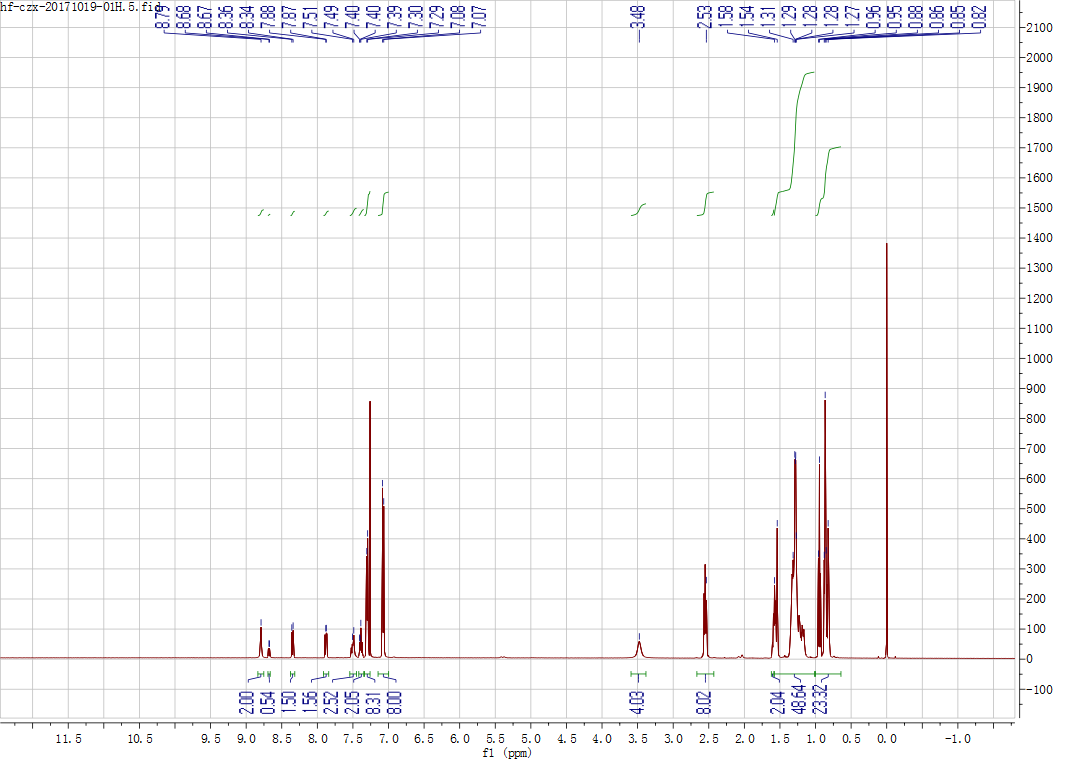


**Figure S8**. ^1^H NMR spectrum of BT-F.


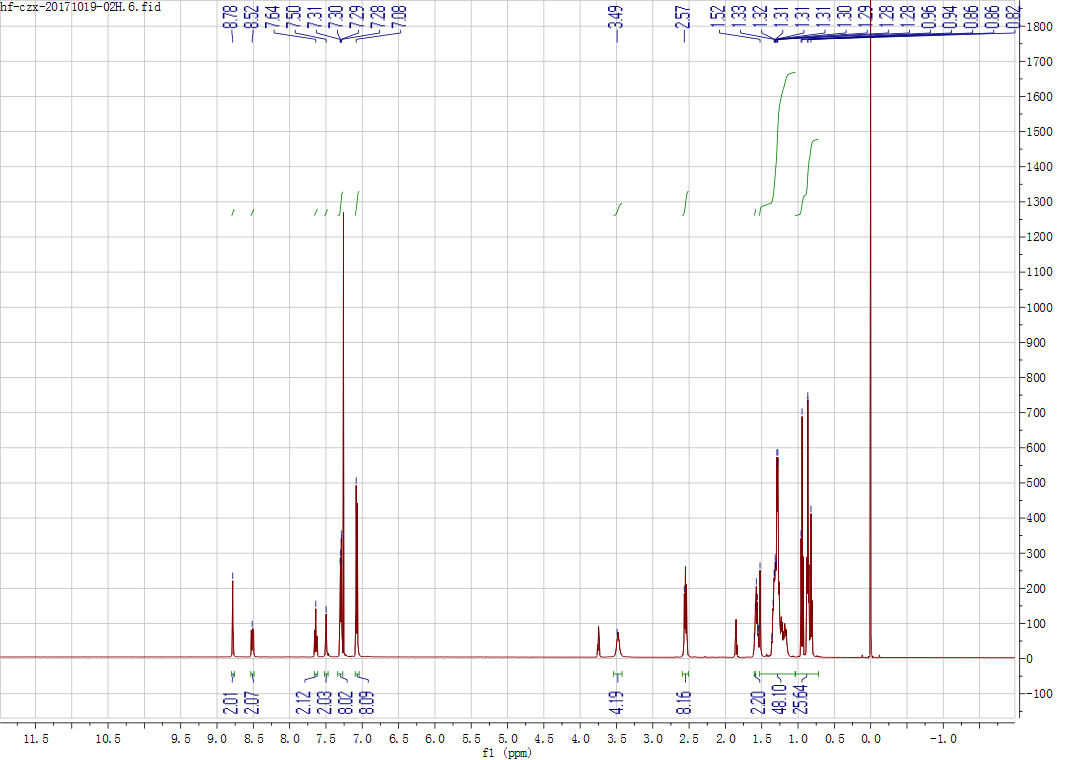


**Figure S9**. ^1^H NMR spectrum of BT-2F.


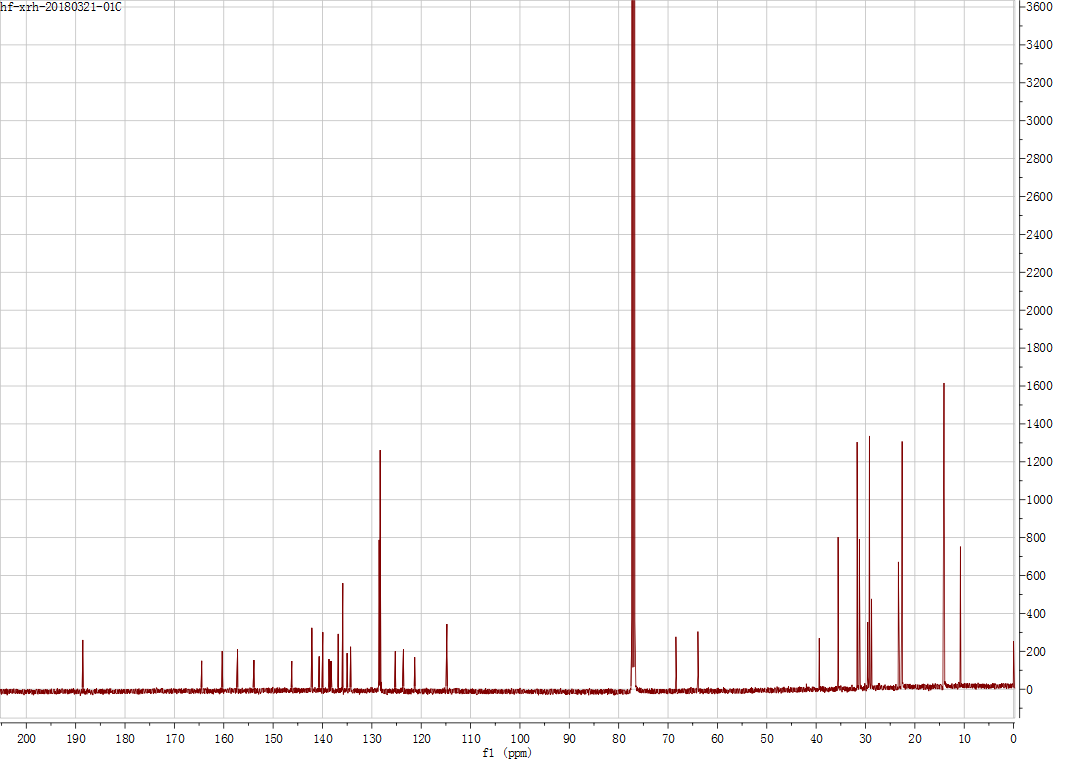


**Figure S10**. ^13^C NMR spectrum of BT-IC.


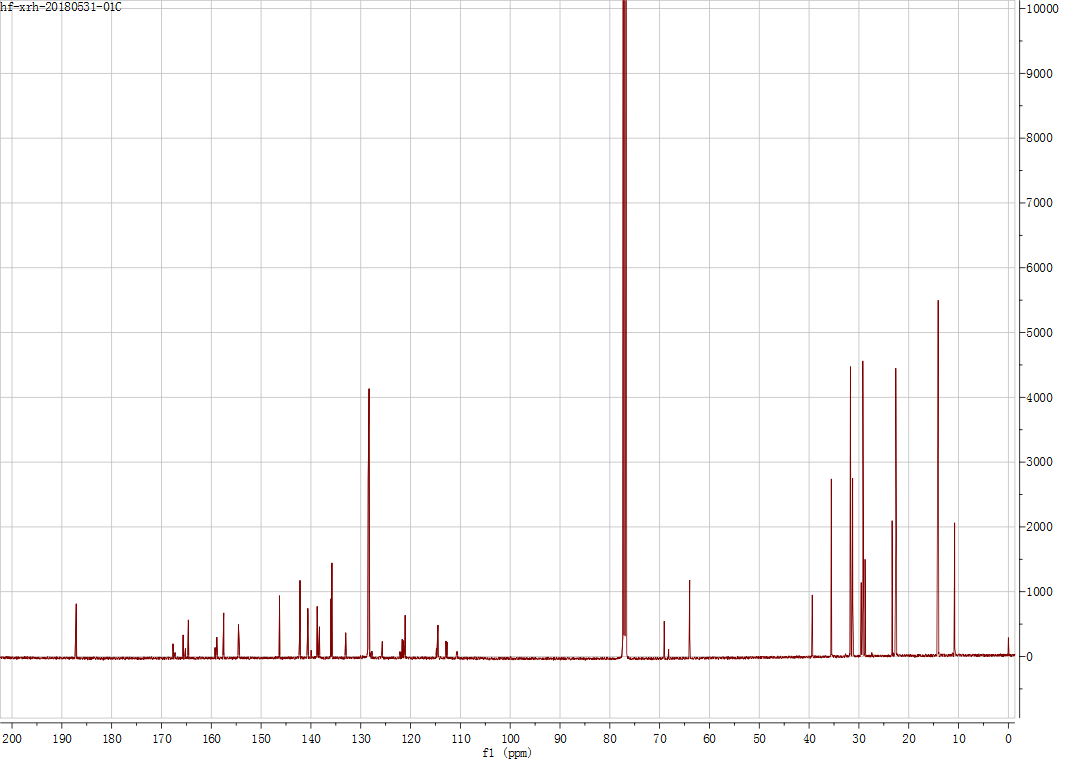


**Figure S11**. ^13^C NMR spectrum of BT-F.


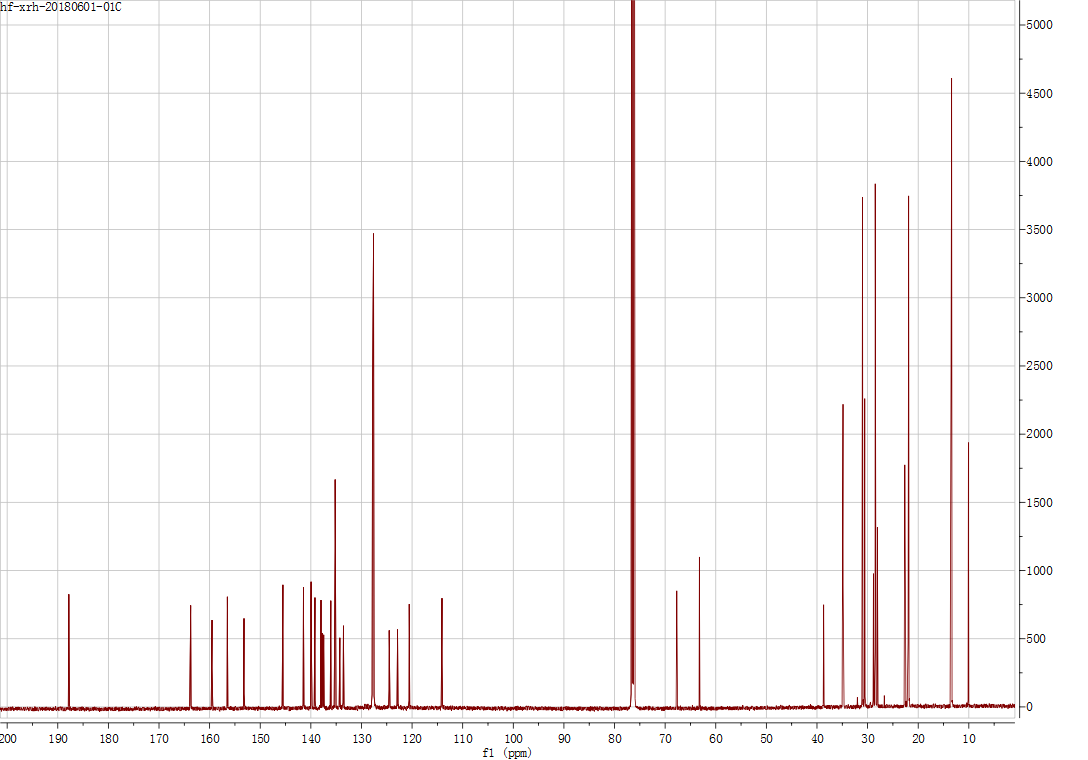


**Figure S12**. ^13^C NMR spectrum of BT-2F.
